# A keep‐it‐simple embolisation approach to treat pelvic congestion syndrome without compromising clinical effectiveness

**DOI:** 10.1111/1754-9485.13795

**Published:** 2024-10-20

**Authors:** Eisen Liang, Wai Yan Timothy Wong, Razeen Parvez, Michael Chan, Bevan Brown

**Affiliations:** ^1^ Sydney Fibroid Clinic Sydney New South Wales Australia; ^2^ Gosford Hospital Gosford New South Wales Australia; ^3^ Sydney Adventist Hospital Sydney New South Wales Australia

**Keywords:** ovarian vein embolisation, pelvic congestion syndrome, venous embolisation

## Abstract

**Introduction:**

There are two approaches to treating pelvic congestion syndrome (PCS): (i) the keep‐it‐simple (KIS) approach, which involves embolising only the refluxing vein(s), typically the left ovarian vein (LOV) unless the right ovarian vein (ROV) or left/right internal iliac vein (IIV) tributaries are also refluxing; and (ii) the extensive (EXT) approach, which empirically embolises almost all of the LOV, ROV, and left and right IIV tributaries. The aim of this study is to determine whether the KIS approach can effectively treat PCS while minimising the number of treated veins and coils used, without the need for injecting sclerosing agents into pelvic veins or the use of occlusion balloons.

**Methods:**

This is a single‐institution retrospective cohort study. Our records identified 154 women who underwent venograms for possible PCS, with the intent to proceed with embolisation. Refluxing veins were treated using the KIS approach, deploying minimal number of coils, ‘sandwiching’ sclerosing foam. Short‐term follow‐up was conducted at 6 weeks; long‐term follow‐ups (between 12 and 60 months) were conducted via an electronic survey consisting of 19 questions assessing pelvic pain/pressure, leg and back pain, fatigue, and bladder and menstrual symptoms.

**Results:**

Fifteen women had negative venogram; 139 women had one or more refluxing veins on venogram. Most women (73%) required unilateral ovarian vein (OV) embolisation, 14% required bilateral OV embolisation, and 12% underwent pelvic vein embolisation. Most cases required only four pushable coils. Clinical success was 89% at 6 weeks and 84% at 1–5 years. A visual analogue scale reduction of 5.2 points (from 7.8 to 2.7) was achieved. There were no instances of coil dislodgement or other complications.

**Conclusion:**

The keep‐it‐simple approach, embolising only the refluxing ovarian and/or iliac veins, can achieve a successful clinical outcome for pelvic congestion syndrome. The extensive approach of empirically embolising all ovarian veins and internal iliac veins may not be necessary. This carries implications for potential savings in procedure time, cost and radiation dose.

## Introduction

Chronic pelvic pain in women, caused by pelvic venous congestion (PCS), can be treated with venous embolisation.^1^ There are two divergent philosophical approaches to conduct the procedure: the keep‐it‐simple (KIS) approach—embolising only the refluxing vein(s), typically only the left ovarian vein (LOV), unless the right ovarian vein (ROV), left or right internal iliac vein (IIV) tributaries are also refluxing^2^, ^3^—and the extensive (EXT) approach—empirically embolising almost all of the LOV, ROV, and left and right IIV tributaries.^4^, ^5^

Previous studies comparing the EXT and KIS approaches have reported similar clinical outcomes.^1^ The KIS approach could be associated with shorter procedure times, reduced radiation exposure, fewer coils, and theoretically a lower risk of coil migration. Considering the prevalence of PCS, there is potential for reducing healthcare costs by using the KIS approach. However, a recent systematic review on clinical outcomes seems dominated by the EXT approach, with a large number of ROVs and IIVs embolised to treat PCS.^6^ The aim of this study is to determine if the KIS approach is sufficient to effectively treat PCS, embolising only refluxing veins.

## Methods

The study was approved by the local research ethics committee.

### Patient recruitment

This is a retrospective analysis of all patients undergoing venogram and embolisation for PCS in a single centre over a five‐year period between 2018 and 2022. Local ethics approval was obtained in 2022. The electronic records from a women's health interventional radiology clinic were searched, to identify women who had undergone endovascular management for possible PCS.

Clinical inclusion criteria for venogram are convincing symptoms of PCS (e.g. dull aching pelvic pain or discomfort towards the end of the day or after physical activities, leg pain, pain during and after sexual intercourse), and imaging features of PCS, such as pelvic varicosity, dilated adnexal veins of more than 5 mm, increased flow during Valsalva, or refluxing ovarian vein, on either transvaginal ultrasound (TVUS), contrast‐enhanced CT or magnetic resonance imaging with venogram (MRI/MRV). Previous endovascular treatment of incompetent veins was allowed.

Exclusion criteria for venogram were clinical history and investigations suggesting alternative pathologies as the predominant cause of pelvic pain.

### Venogram and embolisation protocol

All venograms and embolisation procedures were performed by a single operator who has more than 20‐year experience in treating PCS. All procedures were performed under local anaesthetic with conscious sedation (intravenous midazolam and fentanyl), using a right internal jugular vein approach under ultrasound guidance, via a 5Fr sheath and 5Fr diagnostic catheters, such as MP A1 (Cordis Australia). Glyceryl trinitrate 50 mg topical patch was applied 30 min prior to the procedure to prevent venous spasm.

Catheter venography was performed to interrogate the bilateral renal, ovarian and internal iliac veins; external compression of the left renal vein and left common iliac vein was checked for and excluded. With our ‘Keep‐It‐Simple’ (KIS) protocol, only refluxing ovarian and/or internal iliac vein tributaries connected to the pelvic varicosity were embolised. The Valsalva manoeuvre was used to demonstrate reflux unless it was already evident during quiet respiration.

**Fig. 1:**
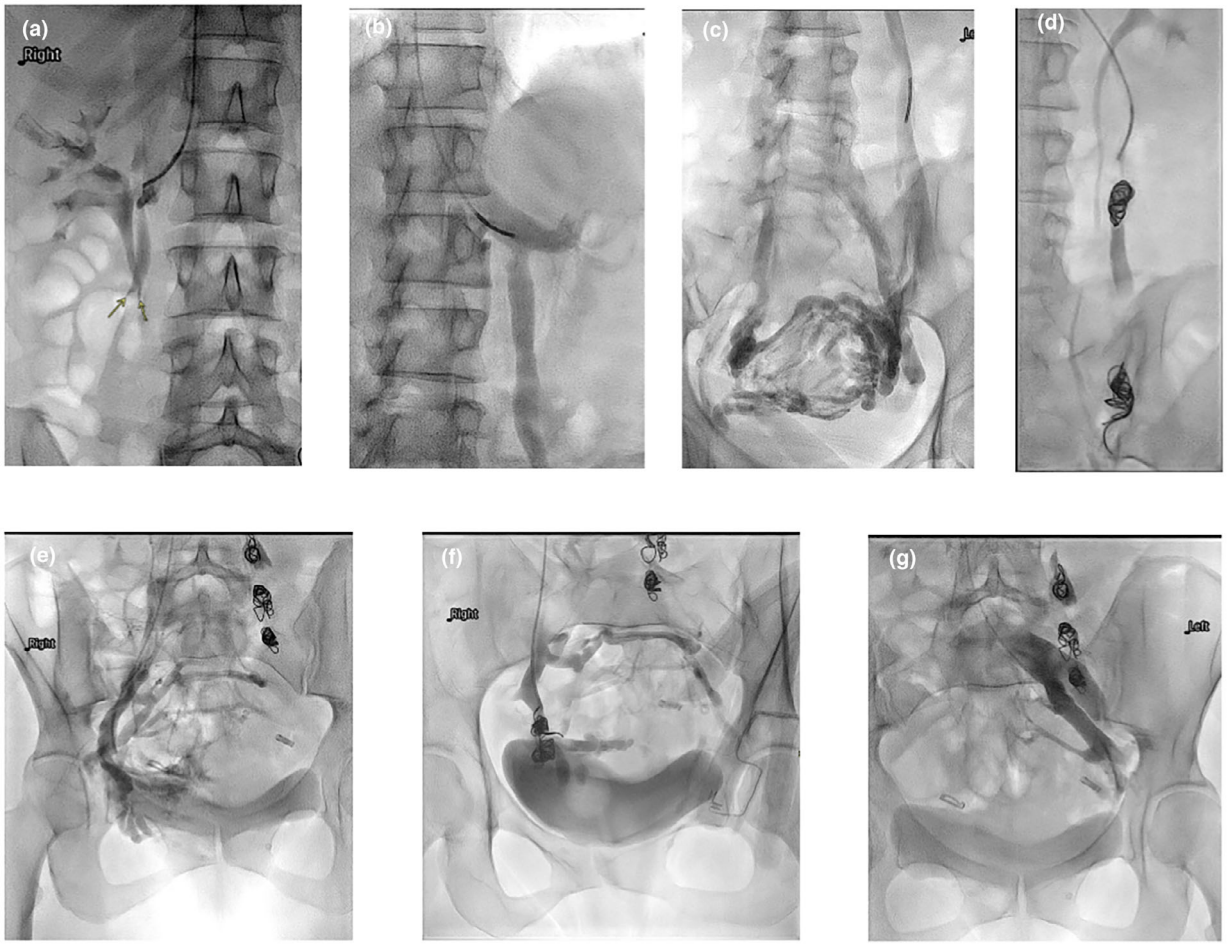
‘Keep‐it‐simple’ embolisation approach. (a) ROV venogram showing intact valve leaflets (arrows) and no reflux, therefore not embolished. (b) Left renal venogram showing readily refluxing LOV. (c) LOV injection showing retrograde flow feeding extensive pelvic varicosities. (d) LOV embolisation using the ‘Sandwich’ technique with two nests of pushable coils. Note the proximal coils are about 5 cm distal to the level of the renal vein. (e) RIIV venogram showing reflux into pelvic varicosities. (f) Coil embolisation of the confluence of the anterior division tributaries of the RIIV. (g) LIV venogram with a good Valsalva showing no reflux or filling of pelvic varicosities and therefore not embolised.

A ‘Sandwich’ technique similar to that of the testicular vein embolisation described by Reiner^7^ was used to embolise the ovarian vein, deploying a nest of 1–2 pushable coils distally at the pelvic brim level to slow down the flow to near stasis, followed by 1–2 mL of sclerosing foam injected proximal to the coil nest. The catheter was then positioned further proximally, to about 5 cm from its junction with the renal vein on the left, or IVC on the right, and a second nest of 1–2 coils was deployed, followed by 1 mL of sclerosing foam, injected slowly proximal to the coil nest. Any significant ovarian vein branches were either individually embolised or their junctions covered by coils and sclerosing foam. Any refluxing internal iliac vein anterior division tributaries feeding the pelvic varicosity were also embolised, typically using only one nest of coils and optional sclerosing foam just proximal to the coil nest, if deemed safe to do so (Fig. 1).

Pushable coils used were fibred platinum, 14 cm long (Nester Coils; COOK Australia), sized at 20% larger than the measured diameter of the target vein. Retractable coils were rarely used, only when the operator felt unsafe to use pushable coils due to the unfavourable anatomy of the target vein. The sclerosing foam was prepared by mixing 4 mL air with 2 mL 3% sodium tetradecyl sulfate (Fibro‐Vein; Australasian Medical and Scientific Limited) or 3% polidocanol (Aethoxysklerol; Getz Healthcare Pty Ltd, Australia), agitated 20 times through a three‐way stopcock using two 5 mL syringes, similar to the technique originally described by Tesarri.^8^

Out KIS approach did not involve injecting sclerosing agents into the pelvic veins or use of occlusion balloons.

### Short and long‐term follow‐up

Short‐term clinical follow‐up was conducted at 6 weeks by clinic visit or telephone follow up. Short‐term success was defined as if the women reported ‘Complete or Significant improvement of symptoms’ and ‘Happy or Very happy’ with the outcome.

At the time of the study, women who had embolisation more than 12 months ago were identified and an electronic survey consisting of 19 questions (Appendix I) were sent to them. Pelvic pain using a visual analogue scale (VAS) at baseline and follow‐up was recorded, and the reduction in VAS was calculated. Overall long‐term clinical success was defined as if the women answered ‘Very Happy/Very Satisfied’ or ‘Happy/Satisfied’. Failure was defined as if women answered ‘Not sure’, ‘Unhappy/Unsatisfied’ or ‘Very Unhappy/Very Unsatisfied’.

No all patients attended 6 week follow‐up. Not all patients responded to the long‐term survey. Some attended 6 week follow‐up but did not respond the long‐term survey; some did not attend 6 week follow‐up but responded to long‐term survey. Therefore the short‐term cohort is not identical as the long term cohort.

Changes in other ancillary symptoms, such as pelvic pressure, back pain, leg pain/heaviness, bladder symptoms, pain during sex, pain after sex, heavy menstrual bleeding, period pain, premenstrual pain or bloating, fatigue, and the requirement for pain medications, were surveyed and recorded as ‘All gone’, ‘Significant improvement’, ‘Some improvement’, ‘No change’ or ‘Worsen’.

Women who required repeat venogram and embolisation of additional veins were identified, and their outcomes reported.

### Study objectives and statistical analysis

The primary outcome was to evaluate the short and long term clinical success of the KIS approach for venous embolisation in PCS. The secondary outcomes were to evaluate if demographics (e.g. age, parity), symptomatic length, type of symptom (e.g. back pain, leg pain, bladder symptoms) were factors contributing to clinical success or failure.

The data collected were analysed with SPSS version 24 (IBM Corp., Armonk, NY, USA). Patients' characteristics were reported using frequency and descriptive analyses. Follow‐up was calculated from the date of embolisation to the date of the last follow‐up. A *P*‐value of 0.05 or less was considered significant. Binary logistic regression analysis was performed for factors influencing treatment success or symptomatic return.

## Results

**Fig. 2:**
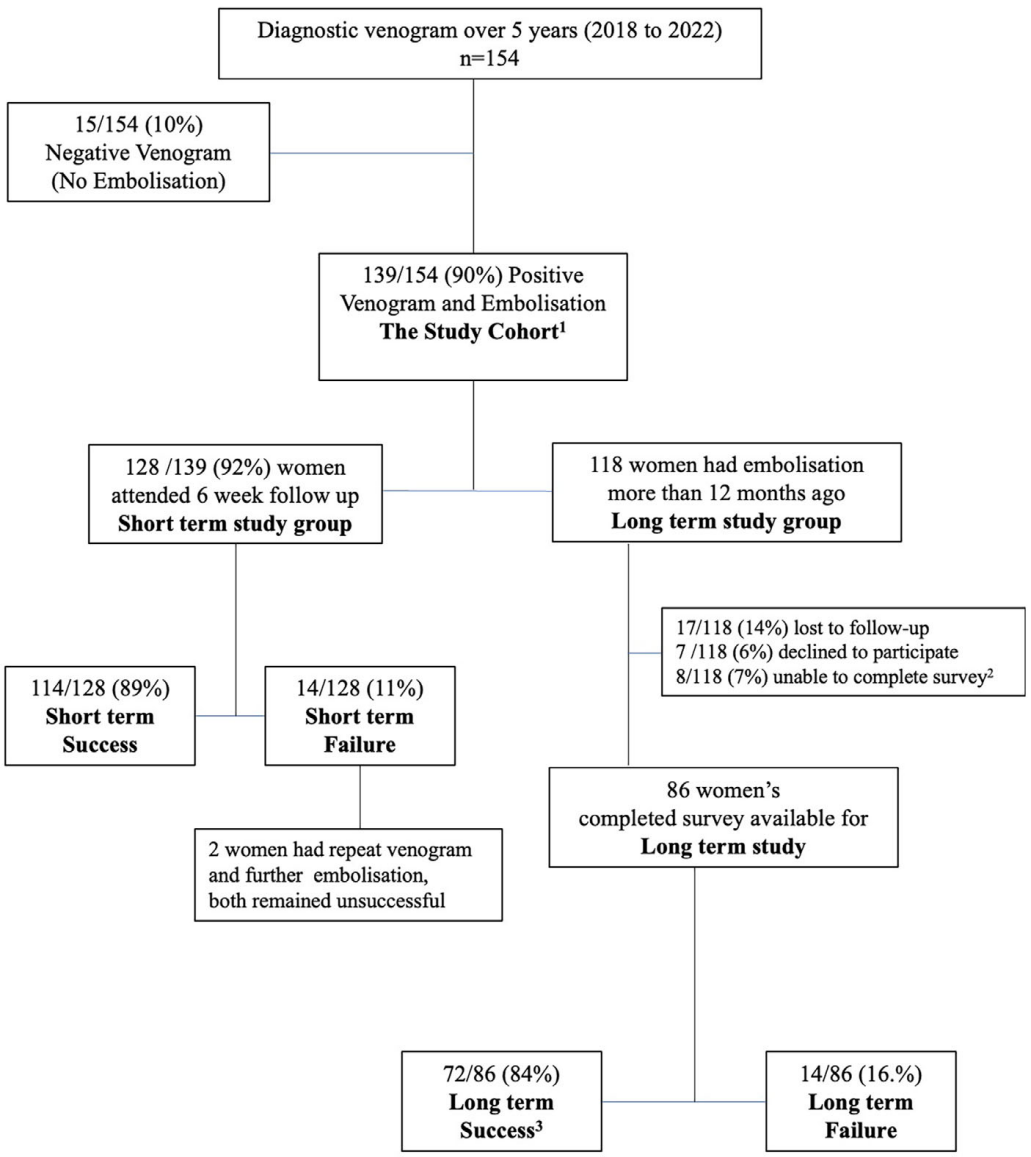
Study cohort. ^1^One woman had prior LOV and Left IIV anterior division embolisation in another institution but remained symptomatic requiring ROV embolisation. ^2^Women contacted the research team or clinic, stating that they were unable to complete the survey, citing concurrent/coexisting/alternative medical conditions (fibromyalgia, Crohn's disease, rectal cancer, pudendal neuralgia, pedunculated fibroid, severe adenomyosis, oversized Mirena, non‐specific bloating). ^3^Including four women has repeat venogram and further embolisation (1 had 2 repeats), all eventually achieved long‐term success.

During the 5‐year study period, 154 women underwent diagnostic venogram, 15 women failed to show any refluxing veins to embolise, and 139 women were found to have one or more refluxing veins and were treated with embolisation (Fig. 2 and Table 1). Of these, 102 (73%) required unilateral ovarian vein embolisation only [71% left ovarian vein (LOV) only; 3% right ovarian vein (ROV) only], 20 (14%) required bilateral ovarian vein embolisation, and 17 (12%) required pelvic vein embolisation (Table 2). The average number of coils used per case was six, with most women requiring only four coils.

**Table 1:** Patient demographics

	Mean	Median	Range
Age	47	45	20–85
Parity	2.5	2	0–7
Pain at baseline (VAS)	7.8	8	0–10
Left ovarian vein diameter	9 mm		
Right ovarina vein diameter	7 mm		
Other pathologies (data obtained from pre‐procedure consultant and survey)			
Adenomyosis		30/139 (22%)	
Lower limb varicose vein		20/139 (14%)	
Endometriosis		3/139 (2%)	
Fibroids		1/139 (0.7%)	
Polycystic ovarian syndrome		1/139 (0.7%)	
Symptoms duration before diagnosis (data obtained from survey)			
<5 months		5/86 (6%)	
6–12 months		18/86 (21%)	
1–2 years		21/86 (24%)	
2–5 years		19/86 (22%)	
More than 5 years		23/86 (27%)	

**Table 2:** Procedure details

	Mean	Median	Mode	Range
No. of coils used per case	6	5	4	3–16
Veins embolised	*n*/139 (%)			
LOV only	98/139 (71%)			
ROV only	4/139 (3%)			
Bilateral OV	20/139 (14%)			
LOV + LIIV	4/139 (3%)			
LOV + RIIV	1/139 (0.8%)			
RIIV only	3/139 (2%)			
LIIV only	1/139 (0.7%)			
LOV + ROV + LIIV	6/139 (4%)			
LOV + ROV + RIIV	1/139 (0.7%)			
LOV + LIIV + RIIV	1/139 (0.7%)			

LIIV, left internal iliac vein; LOV, left ovarian vein; OV, ovarian vein; RIIV, right internal iliac vein; ROV, right ovarian vein.

Six‐week follow‐up were attended by 128/139 (92%) women. Short‐term success was achieved in 114 out of 128 women (89%), who reported being ‘Happy or Very Happy’ with the overall outcome, indicating ‘Complete Resolution’ or ‘Significant Improvement’ of symptoms (Fig. 2).

At the time of study, 118 women were identified to have had embolisation more than 12 months ago (mean 34 months; range: 12–60 months), 101 (86%) women responded the request to long‐term study surveys; 17 (14%) were unable to be reached and lost to follow‐up; 7 (6%) declined to participate; and 8 (7%) were unable to complete the surveys online but contacted the research team or clinic, stating that they were unable to complete the survey, citing concurrent/coexisting/alternative medical conditions with overlapping symptoms (fibromyalgia, Crohn's disease, rectal cancer, pudendal neuralgia, pedunculated fibroid, severe adenomyosis, oversized Mirena, non‐specific bloating). Completed surveys were available for analysis in 86 women (Fig. 2).

Overall, long‐term success was achieved in 72 out of 86 women (84%), who reported being ‘Very Happy/Very Satisfied’ or ‘Happy/Satisfied’ with the overall outcome.

Long‐term outcome of ancillary symptoms is summarised in Table 3. Symptom improvements were noted in high proportion of patients treated, notably: back pain 89%, leg pain/heaviness 90%, bladder symptoms in 74%, pain during sex in 79%, heavy menstrual bleeding 76% and period pain in 79%.

**Table 3:** Long term clinical outcomes of ancillary symptoms

Ancillary symptoms	No of women with symptoms at baseline, *n*/86 (%)	‘All symptoms gone’ *x*/*n* (%)	‘Significant improvement’ *y*/*n* (%)	‘At least some improvement’† *z*/*n* (%)
Pelvic pressure	81/86 (94)	34/81 (42)	32/81 (40)	74/81 (91)
Back pain	52/86 (61)	8/52 (15)	26/52 (50)	46/52 (89)
Leg pain/heaviness	58/96 (67)	15/58 (26)	30/58 (52)	52/58 (90)
Bladder symptoms	54/86 (63)	8/54 (15)	23/54 (43)	40/54 (74)
Pain during sex	52/86 (61)	12/52 (23)	13/52 (25)	41/52 (79)
Pain after sex	53/86 (62)	14/53 (26)	11/53 (21)	41/53 (77)
Heavy menstrual bleeding	49/86 (57)	11/49 (22.4)	14/49 (28.6)	37/49 (76)
Period pain	62/86 (72)	11/62 (18)	21/62 (34)	49/62 (79)
Premenstural pain or bloating	67/86 (78)	10/67 (15)	21/67 (31)	49/67 (73)
Fatique	66/86 (77)	11/66 (17)	22/66 (33)	48/66 (73)
Requirement for pain medications	72/86 (84)	30/86 (42)	20/86 (28)	62/86 (86)

† At least some improvement includes patients with symptoms ‘All gone’; ‘Significant improvement’ and ‘Some Improvement’.

Three women required one repeat venogram and additional embolisation, and one woman required two repeat venograms and further embolisation. These four women eventually achieved long‐term success.

For those who reported symptomatic relief, the majority (77%) experienced relief within 1 month: 17% reported immediate relief, 23% within a week and 30% within a month.

The mean pain reduction was 5.2 on the visual analogue scale (VAS), decreasing from 7.8 ± 1.7 to 2.7 ± 1.7 (*P* < 0.001). Notably, 42% of women no longer required pain medications, and 86% reported a reduced requirement for pain medications. Post‐procedure, eight (6%) patients experienced mild to moderate pelvic or loin pain, attributed to thrombophlebitis. No worsening of pain or pelvic pressure was recorded. Only one patient reported worsening of leg pain/pressure, one reported worsening of period pain, and one reported worsening of heavy menstrual bleeding. There were no cases of infection/haemorrhage, or readmission for unexpected outcomes. There was no coil migration or dislodgement.

When assessing the impact of covariates influencing long‐term clinical success, patients who had pre‐procedure pelvic pressure, back pain, leg pain, bladder symptoms, fatigue, period pain and pre‐menstrual bloating were associated with treatment success. Upon binary logistic regression analysis, none of the covariates remained significant (Table 4).

**Table 4:** Assessment of covariates affecting long term treatment success

Covariate (listed below)	Rate of group A	Rate of group B	*P*‐value	OR (95% CI)	Selected test
Pelvic pressure Yes (A) vs No (B)	68/75	4/11	<0.001	17.00 (3.97–72.77)	Fisher exact
Back pain Yes (A) vs No (B)	43/47	29/39	0.042*	3.71 (1.06–12.96)	Fisher exact
Leg pain/Heaviness Yes (A) vs No (B)	48/52	24/34	0.015	5.00 (1.42–17.61)	Fisher exact
Bladder symptoms Yes (A) vs No (B)	37/40	35/46	0.046*	3.88 (1.00–16.07)	Fisher exact
Fatigue Yes (A) vs No (B)	45/48	27/38	0.007**	6.11 (1.56–23.88)	Fisher exact
Dyspareunia Yes (A) vs No (B)	37/41	35/45	0.15	2.64 (0.76–9.21)	Fisher exact
Heavy menstrual bleeding Yes (A) vs No (B)	33/37	39/49	0.38	2.12 (0.61–7.37)	Fisher exact
Period pain Yes (A) vs No (B)	45/50	27/36	0.063	3.00 (0.91–9.89)	Chi‐square
Premenstrual bloating Yes (A) vs No (B)	47/49	25/37	0.001***	11.28 (2.34–54.42)	Fisher exact
Medications needed Yes (A) vs No (B)	58/62	14/24	<0.001***	10.36 (2.83–37.93)	Fisher exact
Age 45 or more Yes (A) vs No (B)	40/45	32/41	0.17	2.25 (0.69–7.38)	Chi‐square
Parity 3 or more Yes (A) vs No (B)	29/36	43/50	0.50	0.67 (0.21–2.13)	Chi‐square
Ovarian vein diameter ≥10 mm Yes (A) vs No (B)	38/43	34/43	0.24	2.01 (061–6.59)	Chi‐square

* *P* ≤ 0.05; **P ≤ 0.01; ***P ≤ 0.001.

When assessing the impact of covariates influencing long‐term symptomatic return, patients without fatigue (34% vs 15%, *P* = 0.032) and patients aged ≥45 (37% vs 11%, *P* = 0.005) were more likely to experience symptomatic return. Patients with bladder symptoms were also more likely to have symptom return, though this did not meet statistical significance (30% vs 15%, *P* = 0.091). Upon binary logistic regression analysis, fatigue (*P* = 0.02) and age ≥45 (*P* = 0.001) remained significant (Table 5).

**Table 5:** Assessment of covariates affecting long‐term symptom return

Covariate (listed below)	Rate of group A	Rate of group B	*P* value	OR (95% CI)	Selected test
Pelvic pressure Yes (A) vs No (B)	16/75	4/11	0.272	0.48 (0.12–1.82)	Fisher exact
Back pain Yes (A) vs No (B)	10/47	10/39	0.798	3.71 (1.06–12.96)	Chi‐square
Leg pain/Heaviness Yes (A) vs No (B)	13/52	7/34	0.636	1.286 (0.45–3.64)	Chi‐square
Bladder symptoms Yes (A) vs No (B)	6/40	14/46	0.091	0.40 (0.14–1.18)	Chi‐square
Fatigue Yes (A) vs No (B)	7/48	13/38	0.032*	0.33 (0.12–0.93)	Chi‐square
Dyspareunia Yes (A) vs No (B)	9/41	11/45	0.785	0.87 (0.32–2.37)	Chi‐square
Pain post sex Yes (A) vs No (B)	9/41	11/45	0.785	0.87 (0.32–2.37)	Chi‐square
Heavy menstrual bleeding Yes (A) vs No (B)	9/37	11/49	0.84	1.11 (0.41–3.04)	Chi‐square
Period pain Yes (A) vs No (B)	12/50	8/36	0.847	1.11 (0.40–3.06)	Chi‐square
Premenstrual bloating Yes (A) vs No (B)	9/49	11/37	0.22	0.53 (0.19–1.46)	Chi‐square
Medications needed Yes (A) vs No (B)	14/62	6/24	0.81	0.88 (0.29–2.63)	Chi‐square
Age 45 or more Yes (A) vs No (B)	5/45	15/41	0.005**	0.22 (0.07–0.67)	Chi‐square
Parity 3 or more Yes (A) vs No (B)	9/36	11/50	0.745	0.67 (0.21–2.13)	Chi‐square
Ovarian vein diameter 10 mm or more Yes (A) vs No (B)	10/43	10/43	1.00	1.00 (0.37–2.72)	Chi‐square

* *P* ≤ 0.05; **P ≤ 0.01; ***P ≤ 0.001.

## Discussion

This study confirmed that the keep‐it‐simple (KIS) approach was effective in treating pelvic congestion syndrome (PCS), achieving short‐term success in 114 out of 128 women (89%) and long‐term success in 72 out of 86 women (84%).

Based on our KIS experience, 73% women required unilateral ovarian vein embolisation, only 14% women required bilateral ovarian vein embolisation, and only12% women require internal iliac vein tributary embolisation. None of our patients required embolisation of all four sets of veins. Comparing with the EXT approach empirically embolising all four sets of veins, KIS approach implicate potential savings in procedure time, radiation dose and coil cost.

Our KIS approach results compare favourably with the extensive (EXT) approach reported by Kim and Venbrux^4^ on 127 cases over 5 years (1998–2003) with a mean clinical follow‐up of 45 months, achieving a similar 83% improvement. However, in their study, an attempt was made to embolise all four veins in two separate sessions (84% had bilateral ovarian vein embolisation, 85% also had iliac vein embolisation), yet 13% showed no improvement. Furthermore, 4% of women reported worsening of symptoms, possibly due to the occlusion of non‐refluxing drainage pathways and the forced injection of sclerosing agent into pelvic veins, causing pelvic thrombophlebitis. Notably, no worsening of pelvic pain or pressure was recorded in our cohort.

In contrast, using our KIS approach, even though only refluxing veins were selectively embolised, only 4.7% of women required a repeat venogram and embolisation to address residual or recurrent. We avoided unnecessary embolisation of antegrade non‐refluxing pathways.

A study by Laborda *et al*., for ultrasound‐proven PCS treated patients with the EXT approach embolisation, had a total of 179 patients (88.6%) complete the 5‐year follow‐up period, achieving a VAS drop of 6.6 from 7.34 to 0.78. However, there was a long lead time (13.5 ± 1.9 months) for patients with severe pain to see clinical improvement.^5^ In contrast, with our KIS approach, a VAS drop of 5.2 was achieved, and 72% reported symptom relief within 1 month. Immediate relief of pelvic congestion symptoms is expected after embolisation of refluxing veins, unless procedure‐induced pain masks and delays the expected improvement. These divergent results between KIS and EXT could be due to the latter contributing to extensive pelvic thrombophlebitis and occlusion of antegrade non‐refluxing drainage pathways.

Laborda *et al*.^5^ also reported four cases of coil migration (1.9%), considered major complications, with two coils migrating to the right external iliac vein and two to the pulmonary arteries. In comparison, with our KIS approach, only a small percentage (12.2%) of women required iliac vein coil embolisation, which carries a greater risk of coil migration compared to ovarian vein embolisation. We hypothesise that embolising fewer iliac veins and having a more targeted approach for refluxing veins minimises the number of coils and the risk of complications. When we embolised the tributaries of the anterior division of the internal iliac vein (IIV), coils were placed as distally as possible to avoid coil migration. Furthermore, using the ‘Sandwich’ technique for ovarian vein embolisation, we did not need to deploy coils within the proximal 5 cm of the ovarian vein, further reducing the risk of coil mal‐deployment. We also used glyceryl trinitrate (GTN) topical patches to prevent venous spasm, which could predispose improper coil formation in the desired location.

Our study compares similarly to other previous smaller cohort studies utilising the KIS approach to treat PCS. D'Archambeau *et al*.^2^ reported a cohort of 66 patients with a pain scale drop of VAS 5.74 (from 7.88 to 2.15) by using only an average of 4–5 coils. Kwon *et al*.^3^ reported a cohort of 67 patients achieving pain reduction (completely gone or significant reduction) in 82% of cases. Similarly, Kwon also noted relatively early symptom relief within 2–3 months for most patients. This contrasts with Laborda's EXT approach, which required 13.5 months to provide appreciable pain relief.^5^ Kwon's observations support, and our results suggest, that pain symptom relief following embolisation should be almost immediate,^3^ unless complicated by extensive pelvic thrombophlebitis due to indiscriminate embolisation of pelvic veins using the EXT approach.

We propose that PCS in females is a functional analogue to varicocoele in males. We believe women's pelvic varicosities should be treated similarly to men's pampiniform plexus. The common pathophysiology is the incompetence of the gonadal vein, resulting in increased pressure and reversed flow, congesting the pelvic venous plexus in females and the pampiniform plexus in males. When treating male varicocele, one would not contemplate injecting sclerosants into the varicocele itself, as this would cause painful thrombophlebitis. Similarly, the logical approach to treat PCS is to address the root cause of the congestion (the refluxing gonadal veins). This approach will lead to the collapse of the varicosities, which, no longer being subject to the pressure of the refluxing veins, will decompress and will no longer be a cause of symptoms.

Incompetence of iliac vein tributaries can contribute to PCS in females, but published studies utilising the KIS technique suggest that this is uncommon.^2^, ^3^

Venous embolisation by using ethylene vinyl alcohol copolymer (Onyx)^9^ and Amplatzer Vascular Plug (AVP)^10^ have been described. There is no comparison study to show that these more complicated and expensive devices has a better outcome than pushable coils.

This present study has some limitations. First, our follow‐up is relatively short, ranging between 12 and 60 months. However, in common with Kwon,^3^ we have noted that most patients report relief within 1–3 months, and therefore a follow‐up study of treated PCS patients for a minimum of 12 months should be sufficient. Second, results from this single‐centre, single‐operator cohort study may not translate to worldwide practice. However, with a consistent technique and strict adherence to protocol, the results can be a useful reference benchmark for future studies. Finally, our outcome survey questionnaire has not been validated, and retrospectively collected data suffers inherent biases. Ideally, to formally validate the KIS approach, a prospective randomised comparison study comparing KIS and EXT approaches would be required.

In conclusion, the keep‐it‐simple approach, embolising only the refluxing ovarian and/or iliac veins, can achieve a successful clinical outcome for pelvic congestion syndrome. The extensive approach of empirically embolising all ovarian veins and internal iliac veins may not be necessary. This carries implications for potential savings in procedure time, cost and radiation dose.

## Funding

There was no financial support from outside organisations.

## Declaration

We declare that all procedures performed were in accordance with the ethical standards of the institutional and/or national research committee and with the 1964 Helsinki Declaration and its later amendments or comparable ethical standards.

## Consent to participate

Informed consent was obtained from all individual participants involved in the study.

## Data Availability

Data are available for review upon request.

## Appendix I

### Post‐procedure survey

Thank you for agreeing to participate in this audit program. The questions are related to the common symptoms of pelvic congestion syndrome.

We would like to see how some of these symptoms have changed after your OVE procedure.

Please make sure that you click the SUBMIT button at the end of the questionnaire.

Please indicate the changes of the following symptoms:

Q1. Worst pelvic pain before procedure on the scale

0 – 1 – 2 – 3 – 4 – 5 – 6 – 7 – 8 – 9 – 10

Q2. Worst pelvic pain now after procedure

0 – 1 – 2 – 3 – 4 – 5 – 6 – 7 – 8 – 9 – 10

Q3. Pelvic pressure

Nil before/All gone/Significant improvement/Some improvement/No change/Worse

Q4. Back pain

Nil before/All gone/Significant improvement/Some improvement/No change/Worse

Q5. Leg pain or heaviness

Nil before/All gone/Significant improvement/Some improvement/No change/Worse

Q6. Bladder symptoms

Nil before/All gone/Significant improvement/Some improvement/No change/Worse

Q7. Fatigue

Nil before/All gone/Significant improvement/Some improvement/No change/Worse

Q8. Pain during sex

Nil before/All gone/Significant improvement/Some improvement/No change/Worse

Q9. Pain after sex

Nil before/All gone/Significant improvement/Some improvement/No change/Worse

Q10. Heavy menstrual bleeding

Nil before/All gone/Significant improvement/Some improvement/No change/Worse

Q11. Period pain

Nil before/All gone/Significant improvement/Some improvement/No change/Worse

Q12. Premenstrual pain or bloating

Nil before/All gone/Significant improvement/Some improvement/No change/Worse

Q13. Requirement for pain medications

Nil before/All gone/Significant improvement/Some improvement/No change/Worse

Q14. Other discomfort

Nil before/All gone/Significant improvement/Some improvement/No change/Worse

Q15. Have you had any other procedures since your OVE with Dr Liang?

Nil before/Repeat venous embolisation/UAE/Laparoscopy/Others: Free text

Q15a. Other—Comment

Free text

Q16. In terms of overall outcome from the procedure, are you

Very Happy satisfied/Happy satisfied/Not sure/Unhappy unsatisfied/Very unhappy unsatisfied

Q17. After the procedure, how long did it take for symptoms relief to occur?

Immediate/Within a week/Within a month/within 3 months/No relief

Q18. Did the symptoms come back?

Yes/No

Q18a. If yes, after how long? Year(s)

Free text

Q18a. Month(s)

Free text

Q19. How long have you been suffering from symptoms prior to the diagnosis of pelvic congestion?

Less than 6 months/6 to 12 months/1–2 years/2–5 years/More than 5 years

Q20. Do you want to tell the team anything else?

Free text
